# Supplementary material for: Cohesin cleavage by separase is enhanced by a substrate motif distinct from the cleavage site
Source: Nat Commun. 2019 Nov 15;10:5189. doi: 10.1038/s41467-019-13209-y (PMC6858450; doi:10.1038/s41467-019-13209-y)
Supplement: Supplementary file 1 — Supplementary Information [file 41467_2019_13209_MOESM1_ESM.pdf]

Supplementary Information

Cohesin cleavage by separase is enhanced by a substrate motif distinct from the cleavage site

Laura E. Rosen, Joseph E. Klebba, Jonathan B. Asfaha, Chloe M. Ghent,
Melody G. Campbell, Yifan Cheng, and David O. Morgan

a

MTNTAKILNFGRWSHPQFEKGSAGSAAGSGAGWSHPQFEKGSASMTEKTVKAKSSVPASDDAYPEIEKFF
PFNPLDFESFDLPEEHQIAHLPLSGVPLMILDEERELEKLFQLGPPSPVKMPSPPWESNLLQSPSSILST
LDVELPPVCCDIDIGGSGGSGGGSGGGSGENLYFQGDYKDHDGDYKDHDIDYKDDDDKSGP

MRSFKRVNFGTLLSSQKEAEELLPDLKEFLSNPPAGFPSSRSDAERRQACDAILRACNQQLTAKLACPRH
LGSLLELAELACDGYLVSTPQRPPLYLERILFVLLRNAAAQGSPEATLRLAQPLHACLVQCSREAAPQDY
EAVARGSFSLLWKGAEALLERRAAFAARLKALSFLVLLEDESTPCEVPHFASPTACRAVAAHQLFDASGH
GLNEADADFLDDLLSRHVIRALVGERGSSSGLLSPQRALCLLELTLEHCRRFCWSRHHDKAISAVEKAHS
YLRNTNLAPSLQLCQLGVKLLQVGEEGPQAVAKLLIKASAVLSKSMEAPSPPLRALYESCQFFLSGLERG
TKRRYRLDAILSLFAFLGGYCSLLQQLRDDGVYGGSSKQQQSFLQMYFQGLHLYTVVVYDFAQGCQIVDL
ADLTQLVDSCKSTVVWMLEALEGLSGQELTDHMGMTASYTSNLAYSFYSHKLYAEACAISEPLCQHLGLV
KPGTYPEVPPEKLHRCFRLQVESLKKLGKQAQGCKMVILWLAALQPCSPEHMAEPVTFWVRVKMDAARAG
DKELQLKTLRDSLSGWDPETLALLLREELQAYKAVRADTGQERFNIICDLLELSPEETPAGAWARATHLV
ELAQVLCYHDFTQQTNCSALDAIREALQLLDSVRPEAQARDQLLDDKAQALLWLYICTLEAKIQEGIERD
RRAQAPGNLEEFEVNDLNYEDKLQEDRFLYSNIAFNLAADAAQSKCLDQALALWKELLTKGQAPAVRCLQ
QTAASLQILAALYQLVAKPMQALEVLLLLRIVSERLKDHSKAAGSSCHITQLLLTLGCPSYAQLHLEEAA
SSLKHLDQTTDTYLLLSLTCDLLRSQLYWTHQKVTKGVSLLLSVLRDPALQKSSKAWYLLRVQVLQLVAA
YLSLPSNNLSHSLWEQLCAQGWQTPEIALIDSHKLLRSIILLLMGSDILSTQKAAVETSFLDYGENLVQK
WQVLSEVLSCSEKLVCHLGRLGSVSEAKAFCLEALKLTTKLQIPRQCALFLVLKGELELARNDIDLCQSD
LQQVLFLLESCTEFGGVTQHLDSVKKVHLQKGKQQAQVPCPPQLPEEELFLRGPALELVATVAKEPGPIA
PSTNSAPVLKTKPQPIPNFLSHSPTCDCSLCASPVLTAVCLRWVLVTAGVRLAMGHQAQGLDLLQVVLKG
CPEAAERLTQALQASLNHKTPPSLVPSLLDEILAQAYTLLALEGLNQPSNESLQKVLQSGLKFVAARIPH
LEPWRASLLLIWALTKLGGLSCCTTQLFASSWGWQPPLIKSVPGSEPSKTQGQKRSGRGRQKLASAPLSL
NNTSQKGLEGRGLPCTPKPPDRIRQAGPHVPFTVFEEVCPTESKPEVPQAPRVQQRVQTRLKVNFSDDSD
LEDPVSAEAWLAEEPKRRGTASRGRGRARKGLSLKTDAVVAPGSAPGNPGLNGRSRRAKKVASRHCEERR
PQRASDQARPGPRIMETIPEEELTDNWRKMSFRILEGSDGEDSASGGKTPAPGPEAASGEWRLLELDSSK
KKLPSPCPDKESDKDLGPRLQLPSAPVATGLSTLDSICDSLSVAFRGISHCPPSGLYAHLCRFLALCLGH
RDPYATAFLVTESVSITCRHQLLTHLHRQLSKAQKHRGSLEIADQLQGLSLQEMPGDVPLARIQRLFSFR
ALESGHFPQPEKESFQERLALIPSGVTVCVLALATLQPGTVGNTLLLTRLEKDSPPVSVQIPTGQNKLHL
RSVLNEFDAIQKAQKENSSCTDKREWWTGRLALDHRMEVLIASLEKSVLGCWKGLLLPSSEEPGPAQEAS
RLQELLQDCGWKYPDRTLLKIMLSGAGALTPQDIQALAYGLCPTQPERAQELLNEAVGRLQGLTVPSNSH
LVLVLDKDLQKLPWESMPSLQALPVTRLPSFRFLLSYSIIKEYGASPVLSQGVDPRSTFYVLNPHNNLSS
TEEQFRANFSSEAGWRGVVGEVPRPEQVQEALTKHDLYIYAGHGAGARFLDGQAVLRLSCRAVALLFGCS
SAALAVHGNLEGAGIVLKYIMAGCPLFLGNLWDVTDRDIDRYTEALLQGWLGAGPGAPLLYYVNQARQAP
RLKYLIGAAPIAYGLPVSLR*

b

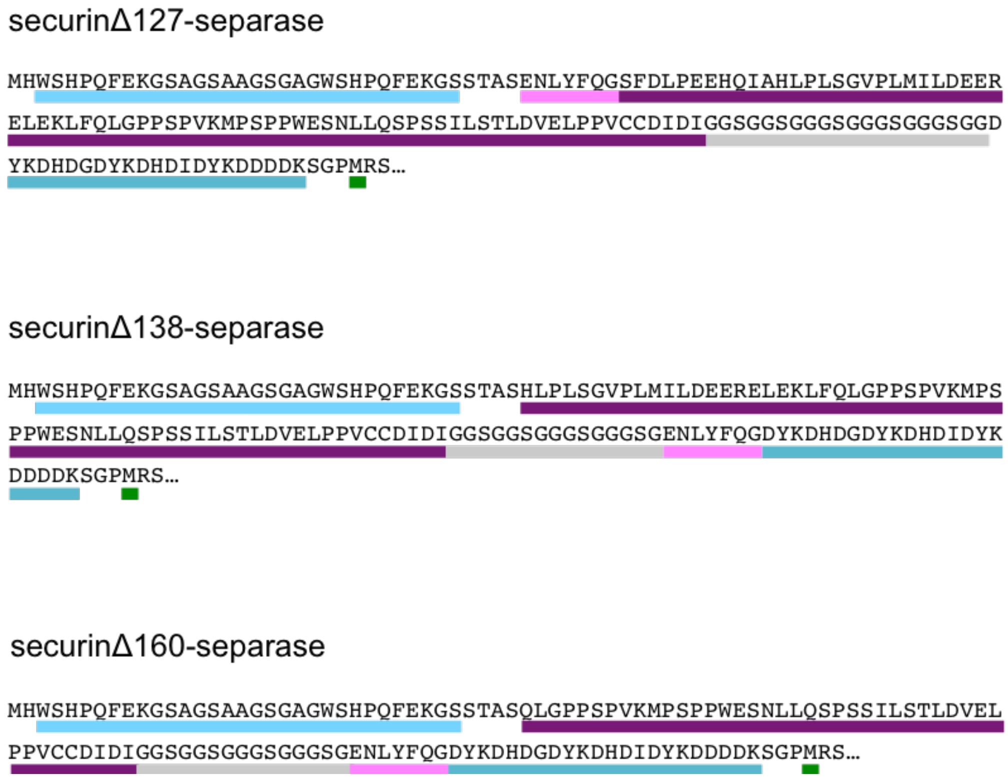

Supplementary Figure 1. Fusion construct sequences

a. Sequence of the securin-separase fusion protein used with TEV protease and ClpXP to generate active separase. Annotated features are: LambdaO ClpXP degron sequence (red), 2x StrepII tag (light blue), securin aa 93-202 (purple), Gly-Ser linker (light gray), TEV protease cleavage site (pink), 3x FLAG tag (teal), Separase ^{1}Met (green), S1126A mutation (red), autocleavage site mutations EXXR → RXXE (dark gray), active site ^{2029}Cys (orange). Dashed gray lines indicate predicted intrinsically disordered regions. The catalytically dead variant is the same sequence except that autocleavage sites are not mutated and ^{2029}Cys is mutated to Ser.

b. Sequences of the securinΔ-separase constructs from the N-terminus to the beginning of separase, after which the sequences are the same as that of separase in panel **a**. Annotations are the same as in panel **a**, except that securin starts at residues 127, 138, or 160. Note that cleavage at the TEV protease site is not necessary for activity toward peptide substrate. For the securinΔ127-separase construct, we were concerned that securin is truncated so close to the pseudosubstrate motif that the adjacent StrepII tag might interfere with the active site; thus, for this construct only we placed the TEV protease site between the 2x StrepII tag and the beginning of securin to allow removal of the tag if necessary. However, activity proved to be normal in the presence of the tag.

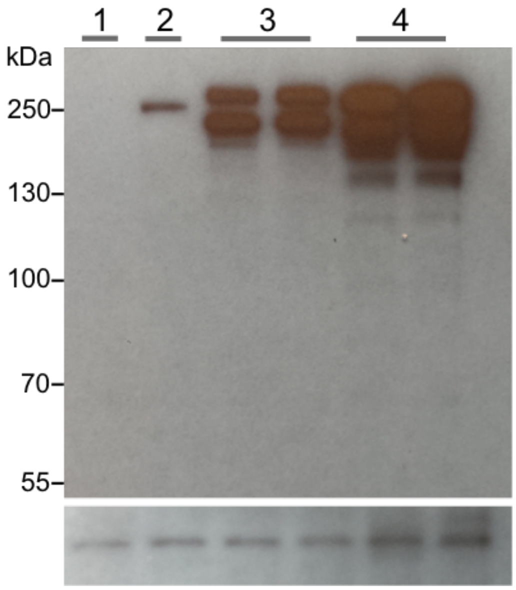

Supplementary Figure 2. Securin-separase expression is enhanced by covalent fusion as compared to co-expression

Sf9 cells were infected with recombinant baculoviruses, and cell lysates were analyzed by SDS-PAGE and Western blotting with antibody against the N-terminal StrepII tag. Lane 1: Uninfected cells; Lane 2: co-infection with two viruses, one encoding securin and the other StrepII-tagged separase C2029S; Lanes 3: two viral isolates encoding securin-separase fusion proteins containing full-length (aa 1-202) securin; Lanes 4: two viral isolates encoding securin-separase covalent fusion proteins containing aa 93-202 of securin, as in Supplementary Fig. 1a. Bottom panel shows a background band (~ 65 kDa) that can be seen when the same blot is overexposed and serves as a loading control. In these pilot studies, the fusion proteins contained an intact active site and wild-type autocleavage sites, resulting in partial autocleavage. Uncropped western blot is provided in the Source Data file.

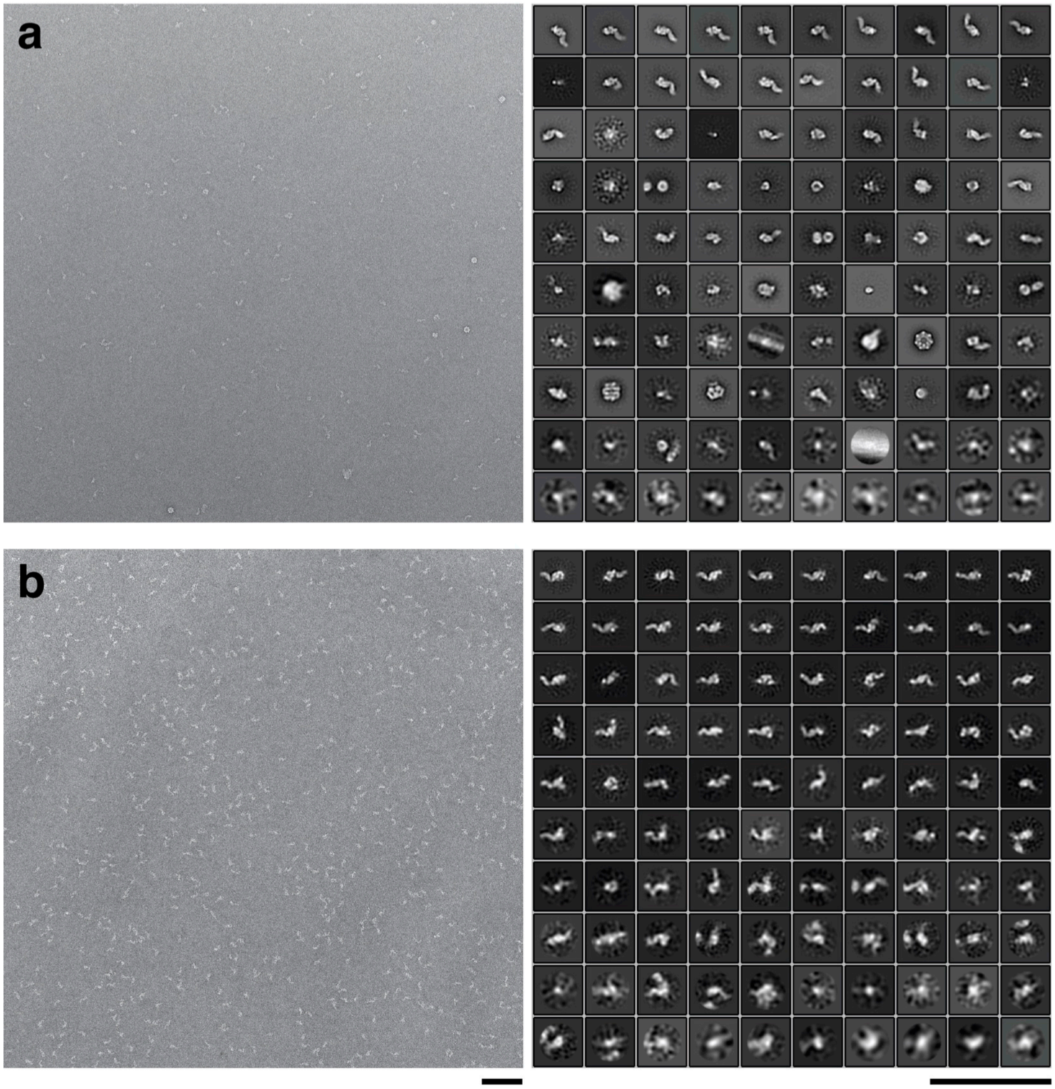

Supplementary Figure 3. Separase electron microscopy data collection and 2D processing
Left: Representative negative stain micrograph of (a) separase alone and (b) securin-separase complex (Scale bar: 100 nm). Right: 2D class averages from 25 iterations of reference-free 2D alignment and classification into 100 classes using all particle picks (Scale bar: 100 nm). A small amount of ClpXP is visible in the separase alone sample, as well as what appear to be separase fragments.

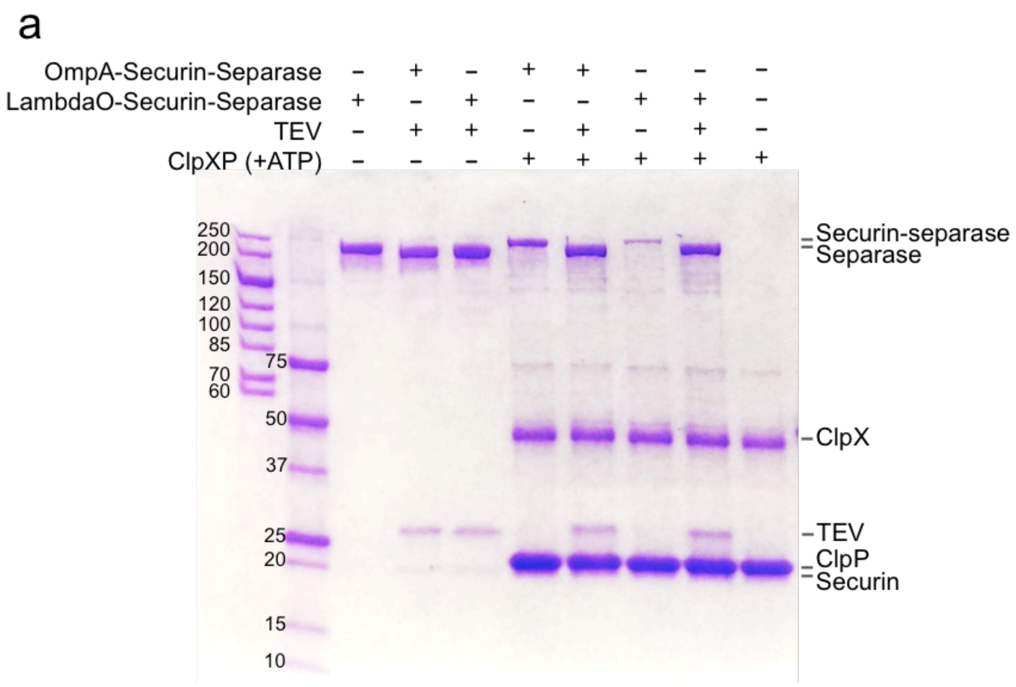
a
OmpA-Securin-Separase
LambdaO-Securin-Separase
TEV
ClpXP (+ATP)
250
200
150
120
100
85
70
60
75
50
37
25
20
15
10
Securin-separase
Separase
ClpX
TEV
ClpP
Securin

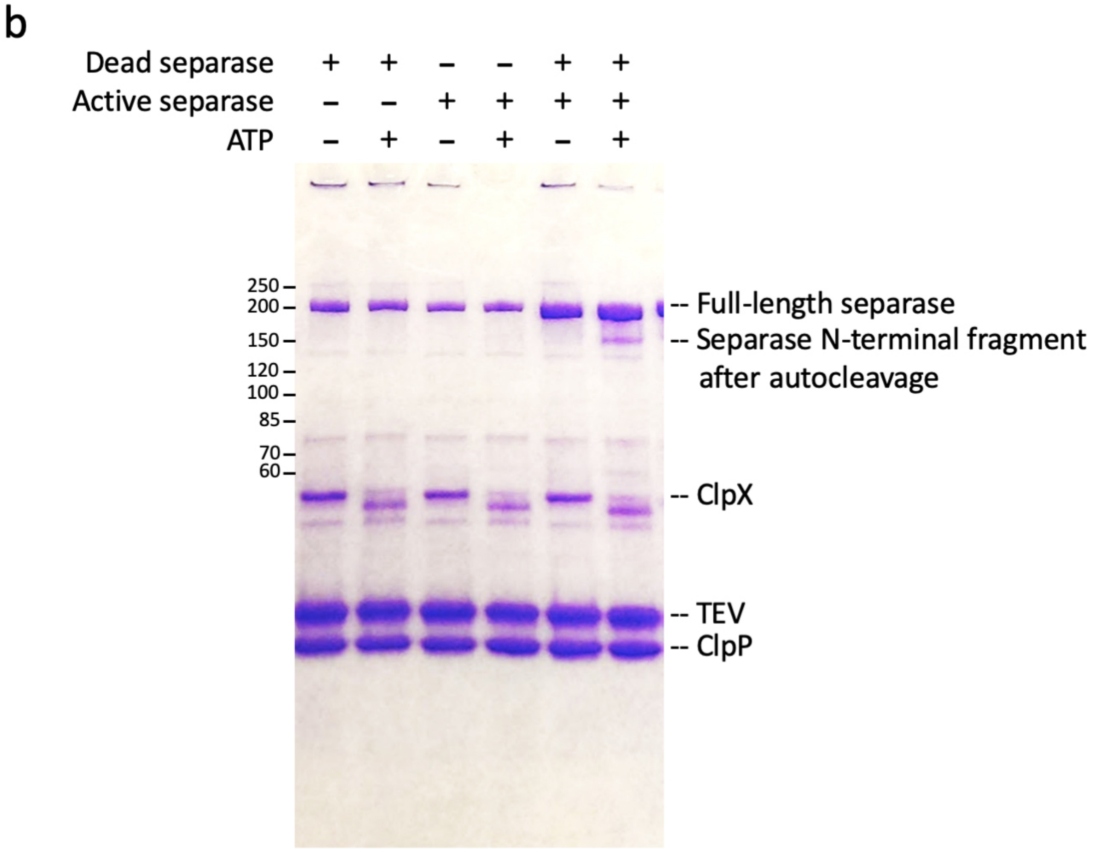
b
Dead separase
Active separase
ATP
250
200
150
120
100
85
70
60
-- Full-length separase
-- Separase N-terminal fragment after autocleavage
-- ClpX
-- TEV
-- ClpP

c

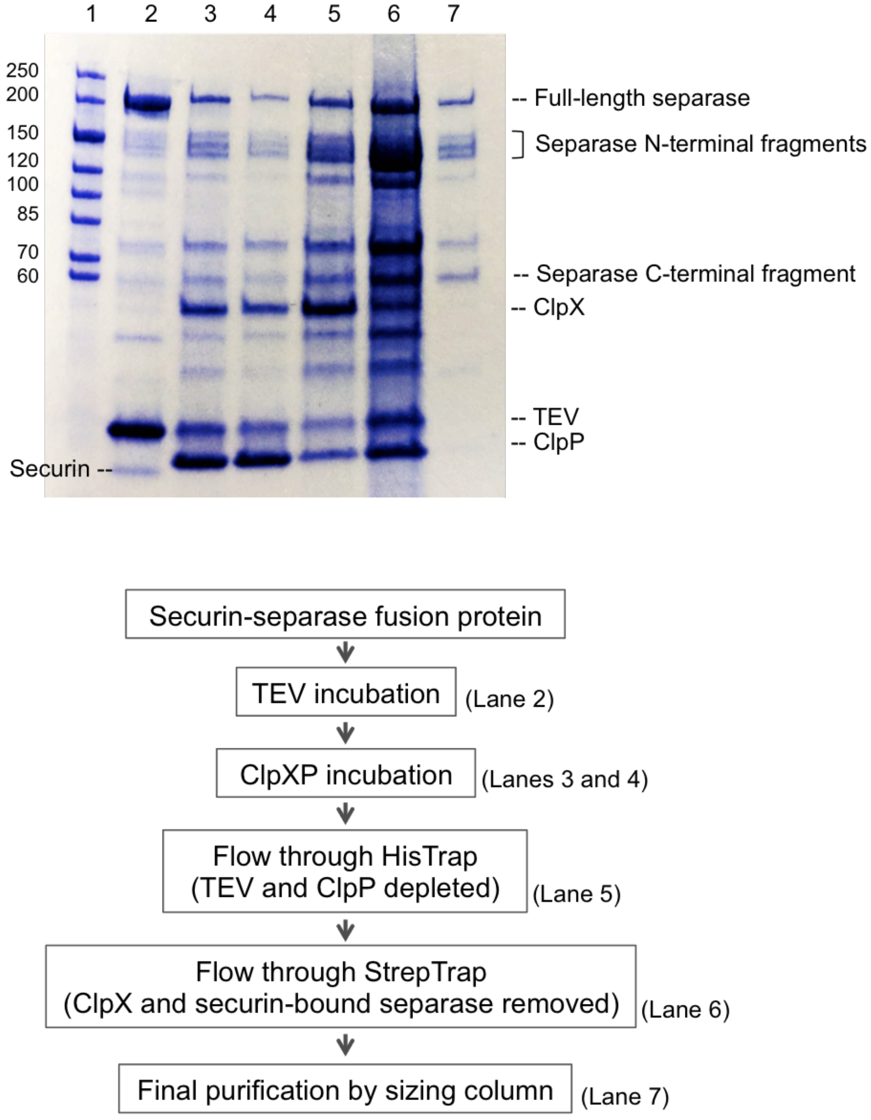
1 2 3 4 5 6 7
250
200
150
120
100
85
70
60
Securin --
-- Full-length separase
Separase N-terminal fragments
-- Separase C-terminal fragment
-- ClpX
-- TEV
-- ClpP
Securin-separase fusion protein
TEV incubation
(Lane 2)
ClpXP incubation
(Lanes 3 and 4)
Flow through HisTrap
(TEV and ClpP depleted)
(Lane 5)
Flow through StrepTrap
(ClpX and securin-bound separase removed)
(Lane 6)
Final purification by sizing column
(Lane 7)

d

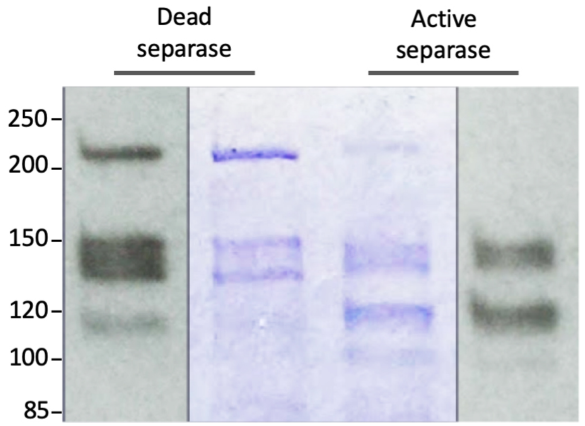
Dead separase
Active separase
250
200
150
120
100
85

e

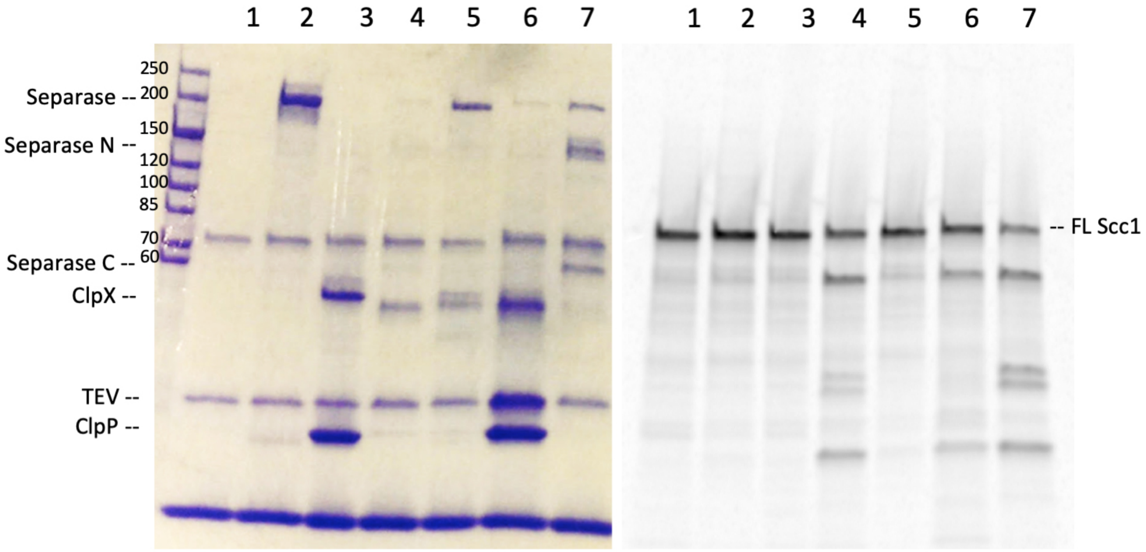

Supplementary Figure 4. Separase activation and purification

a. In these pilot studies, we tested two ClpXP degrons (OmpA and LambdaO). Securin-separase fusion proteins with the indicated ClpXP degron at the N-terminus were incubated with TEV protease followed by ClpXP, or a combination of the two, and subjected to SDS-PAGE and Coomassie Blue staining. TEV protease alone generates a faintly visible securin band. In the absence of TEV protease, ClpXP degrades the securin-separase fusion protein, and greater degradation is seen with the LambdaO degron. After treatment with both TEV protease and ClpXP, ClpXP presumably degrades the cleaved securin, but the securin band, if present, would be obscured by ClpP. Attempts to detect securin by Western blotting were unsuccessful. Based on this and other experiments, LambdaO was found to be the most effective ClpXP degron and was used for subsequent constructs (Supplementary Fig. 1a).

b. Securin-separase fusion proteins (‘Active,’ wild-type active site with mutant autocleavage sites; ‘Dead,’ C2029S mutation with wild-type autocleavage sites) were incubated with TEV protease followed by ClpXP +/- ATP, and subjected to SDS-PAGE and Coomassie Blue staining. Separase activity is apparent as intermolecular cleavage of dead separase in the last lane. The ClpX protein used here undergoes some autodegradation upon activation by ATP.

c. SDS-PAGE and Coomassie Blue staining were used to evaluate the steps in the purification of active separase after TEV protease and ClpXP incubation with the securin-separase fusion protein. Purification steps are listed below the gel. Lanes contain the following: 1. Molecular weight markers (indicated at left in kDa); 2. Fusion protein following incubation with TEV protease; 3. After

incubation with ClpXP (pre-filter); 4. After ClpXP incubation (post-0.2 µm filter); 5. After HisTrap column to remove most TEV protease and ClpP; 6. After StrepTrap column to remove ClpX and securin-bound separase, then concentrated; 7. Active separase after sizing column.

d. To assess the nature of the separase fragments in the preparations, purified separase was evaluated by alignment of the Coomassie-stained gel (inner lanes) with a Western blot against the N-terminal FLAG tag (outer lanes). These results confirm that the smaller bands in the preparation are N-terminal fragments of separase. Because fragments are seen in the dead separase preparation, we conclude that ClpXP is at least partially responsible for this cleavage. Additionally, the amount of full-length separase is reduced in the active separase preparation, which we suspect is due to separase autocleavage at non-canonical sites, presumably an artifact of high enzyme concentration *in vitro*. More full-length separase was preserved in shorter reactions (as seen in panel **c**, lane 7). Despite the extent of separase cleavage into N- and C-terminal fragments as visible by SDS-PAGE, the negative-stain EM results support previous evidence that fragments remain bound together in a structure that is indistinguishable from intact full-length separase at low resolution. Uncropped western blot is provided in the Source Data file.

e. This figure demonstrates that the StrepTrap column separates apo separase from securin-bound separase after TEV and ClpXP incubation. Samples from different steps in the purification procedure were subjected to SDS-PAGE and Coomassie-Blue staining (left) and also tested for activity in the cleavage assay with full-length (FL) Scc1 (right). Lanes: 1. Substrate alone (including TEV protease used for elution of Scc1 from beads, plus unidentified contaminants); 2. Untreated securin-separase fusion protein; 3. ClpXP alone; 4. TEV- and ClpXP-treated separase in flow-through of StrepTrap column, with no securin bound (separase is at low concentration and not clearly visible, but this sample was used for the sizing column whose output is shown in lane 7); 5. TEV- and ClpXP-treated separase eluted from StrepTrap column, with securin still bound; 6. StrepTrap column elution re-treated with ClpXP; 7. Final active separase sample, containing full-length active separase as well as separase N- and C-terminal fragments. In the Scc1 cleavage assay, cleavage occurs primarily at site 1, producing the top and bottom two cleavage fragments; these fragments are not generated when site 1 is mutated (data not shown). Site 2 cleavage would produce two products of similar sizes, but these are not observed in the site 1 mutant. Instead, the high enzyme concentration in some reactions generates two intermediate fragments that probably represent cleavage at a non-canonical site.

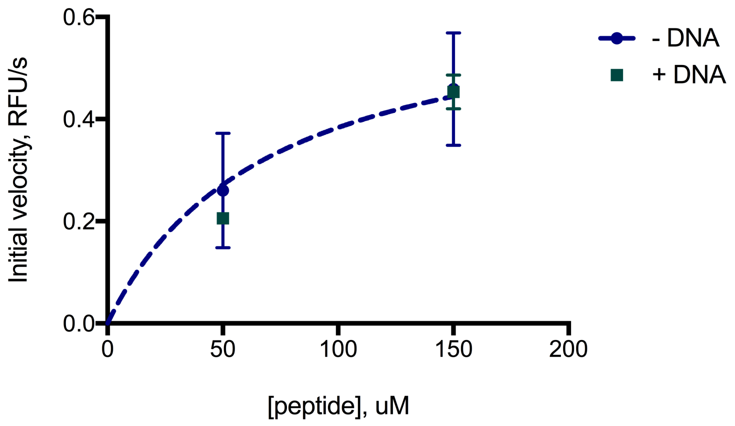

Supplementary Figure 5. Effect of DNA on peptide cleavage by separase

Peptide substrate cleavage rate was measured with purified separase lacking securin, as in Fig. 1h, except that the enzyme was incubated for 30 min with or without 23 µM double-stranded DNA before addition of substrate. DNA was “50 bp #2” (Fig. 1e) but lacking the fluorophore. Data points indicate means (+/- SD) of duplicate samples. For comparison, a Michaelis-Menten curve has been fit to the "-DNA" data points using a K_M value fixed to the value in Fig. 1h (dashed line). Source data are provided in the Source Data file.

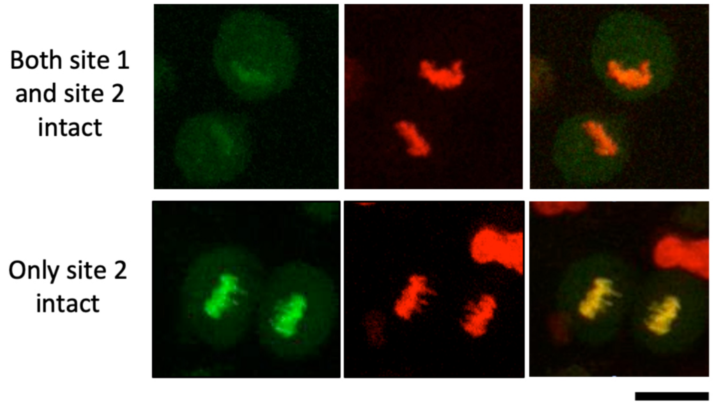

Supplementary Figure 6. Cleavage of Scc1 biosensor is blocked by mutation of site 1 alone

An extended biosensor was created containing Scc1 residues 142-573, either containing wild-type sequence (top) or a mutation at cleavage site 1 (^{169}EXXR to RXXE; bottom). Representative images of late anaphase fluorescence (10 min timepoint) with green fluorescence (left), red fluorescence (center), and merged images (right) show that biosensor cleavage is blocked when site 1 alone is mutated, suggesting that site 2 is not cleaved. Scale bar: 20 µM.

```
        10         20         30         40         50
MATLIYVDKE NGEPGTRVVA KDGLKLGSGP SIKALDGRSQ VSTPRFGKTF

        60         70         80         90        100
DAPPALPKAT RKALGTVNRA TEKSVKTKGP LKQKQPSFSA KKMTEKTVKA

       110        120        130        140        150
KSSVPASDDA YPEIEKFFPF NPLDFESFDL PEEHQIAHLP LSGVPLMILD
                          ^            ^

       160        170        180        190        200
EERELEKLFQ LGPPSPVKMP SPPWESNLLQ SPSSILSTLD VELPPVCCDI
         ^

DI
```

Supplementary Figure 7. Annotated human securin sequence

Yellow indicates APC/C degrons. Red indicates the pseudosubstrate motif, at the start of the separase interaction segment (SIS, underlined). Gray indicates the conserved LPE motif downstream of the pseudosubstrate motif. Arrowheads indicate the first residues in securin-separase fusions (127-202; 138-202; 160-202).

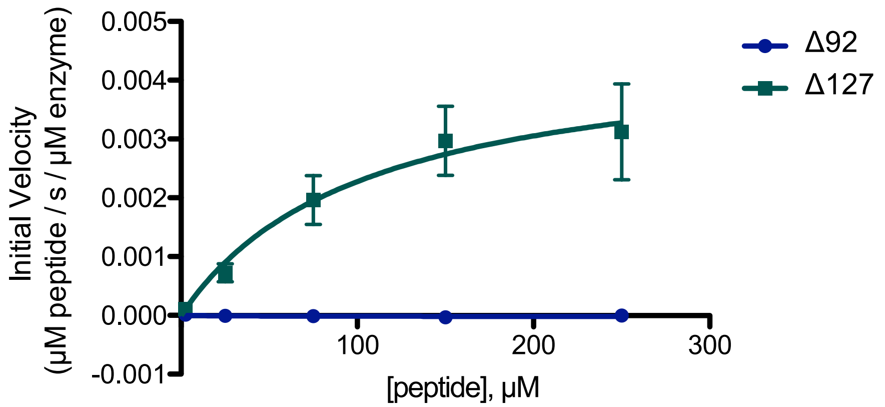

Supplementary Figure 8. Securin pseudosubstrate motif blocks separase activity

Peptide substrate cleavage rate was measured as in Fig. 4c with securinΔ127-separase fusion protein or with the securin-separase fusion containing aa 93-202 of securin (Supplementary Fig. 1a). Initial velocity was normalized by enzyme concentration. Data points indicate means (+/- SD) of triplicate samples. Source data are provided in the Source Data file.

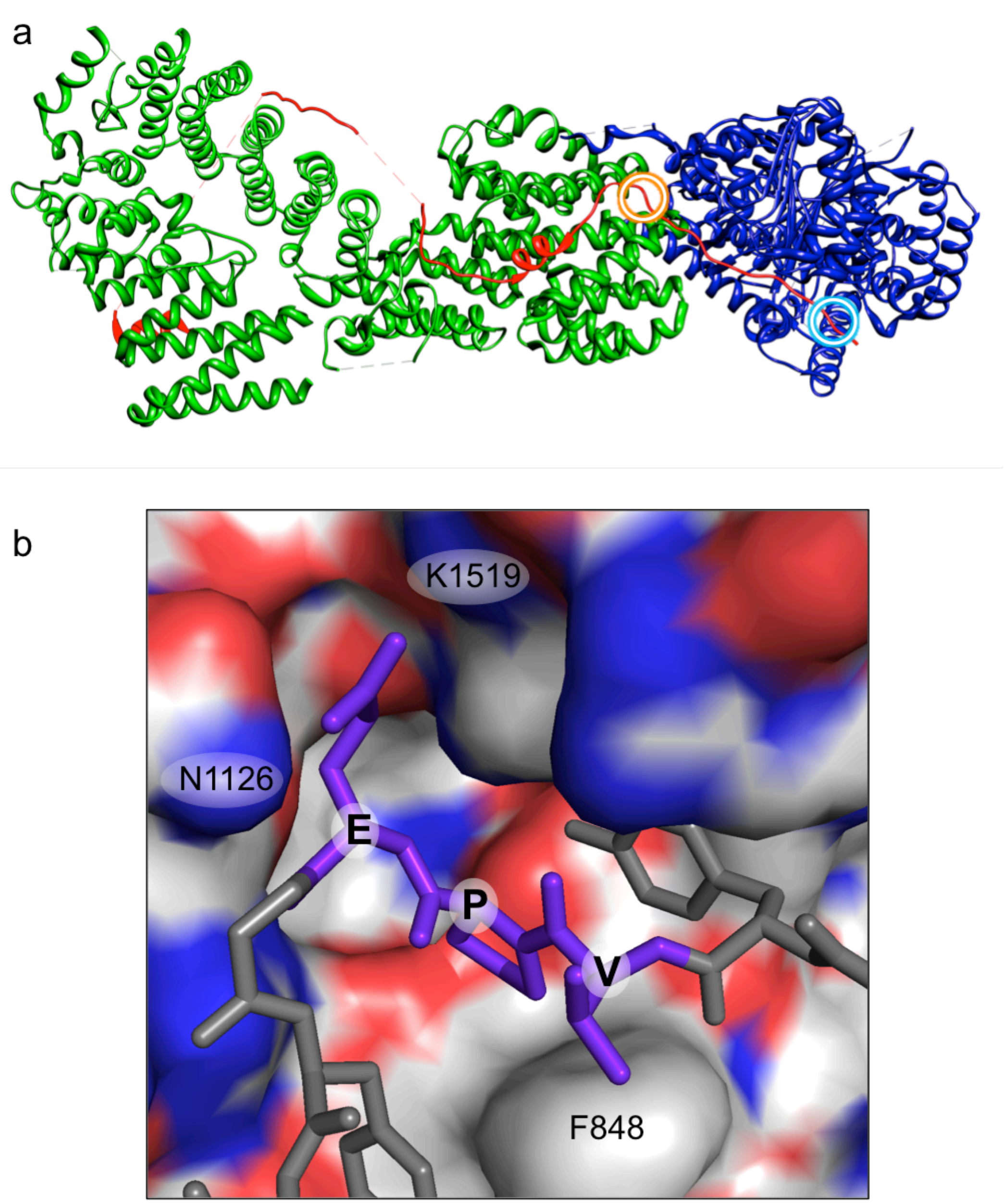

Supplementary Figure 9. Structure of yeast securin VPE motif bound to separase

a. Crystal structure of securin-separase complex from *S. cerevisiae*, with securin in red, the separase protease domain in blue and the remainder of separase in green. Also indicated are the securin VPE motif (orange circle) and securin pseudosubstrate motif in the separase active site (light blue circle). Structure from Luo, S. & Tong, L. *Nature* **542**, 255-259 (2017); PDB: 5u1s.

b. Close-up of the interaction of the securin VPE motif with separase. Separase is illustrated in surface representation colored by charge. Securin is illustrated in gray, with VPE motif highlighted in purple. The side chain of E274 makes hydrogen bonds to separase residues N1126 and K1519, while V272 and P273 are both in van der Waals interaction distance with F848.

a. Vertebrates

Scc1 Site 1

Homo sapiens	167	DREIMREGSA	. . .	248	LSEAGVMLPEQPAHD
Mus musculus	167	DREIMREGSA	. . .	248	LSEAGVMLPEQPAHD
Gallus gallus	167	DREMMREGSA	. . .	247	LSEGGVMMPEQPPHD
Xenopus tropicalis	168	DREMMREGSA	. . .	247	MPEEGVAMPEQPVHD
Dania rerio	167	DREMMREEGA	. . .	250	IPE-GVMMPQDHGAD

Scc1 Site 2

Homo sapiens	445	IEEPSRLQES	. . .	515	LIPELELLPEKEKEK
Mus musculus	449	IEEPSRLQDS	. . .	519	LIPELELLPEKEKEK
Gallus gallus	446	LEEPSRLQES	. . .	517	LIPELELLPEKEKEK
Xenopus tropicalis	448	LEEASRLQES	. . .	528	LIPELNLLPARDRRL
Dania rerio	447	LEEPSVLQAS	. . .	524	LIPDLDLIGEKSKDK

Rec8

Homo sapiens	399	EIEVPREALE	. . .	453	LPVVPELPEVPMEMPL
Mus musculus	449	EIEVLREAQE	. . .	502	LPMLPELPEVPMEMPP
Xenopus tropicalis	444	EVEALRAIAE	. . .	495	LPMVSEMPELMVELPE
Dania rerio	422	SREVIRESEA	. . .	[no LPE motifs]	

b. ***Saccharomyces cerevisiae***

Scc1 site 2	263	SVEQGRRLGE	. . .	369	ITESMSYLPDPILKNF
Rec8 site 2	448	SHEYGRKSFR	. . .	498	ILDFNLNLPPSSFGRS
Slk19	72	SIDYGRSSAL	. . .	161	LKLQLESVPDLKQSST

Supplementary Figure 10. Potential LPE motifs in separase substrates

Vertebrate (**a**) and yeast (**b**) substrate sequences showing cleavage sites (green) and downstream LPE motifs (gray).
