# Supplementary material for: Cohesin cleavage by separase is enhanced by a substrate motif distinct from the cleavage site
Source: Nat Commun. 2019 Nov 15;10:5189. doi: 10.1038/s41467-019-13209-y (PMC6858450; doi:10.1038/s41467-019-13209-y)
Supplement: Supplementary file 3 — Reporting Summary [file 41467_2019_13209_MOESM3_ESM.pdf]

nature research

Corresponding author(s): David Morgan

Last updated by author(s): Sep 1, 2019

Reporting Summary

Nature Research wishes to improve the reproducibility of the work that we publish. This form provides structure for consistency and transparency in reporting. For further information on Nature Research policies, see Authors & Referees and the Editorial Policy Checklist.

Statistics

For all statistical analyses, confirm that the following items are present in the figure legend, table legend, main text, or Methods section.

n/a	Confirmed	
☐	☒	The exact sample size (*n*) for each experimental group/condition, given as a discrete number and unit of measurement
☐	☒	A statement on whether measurements were taken from distinct samples or whether the same sample was measured repeatedly
☒	☐	The statistical test(s) used AND whether they are one- or two-sided<br>*Only common tests should be described solely by name; describe more complex techniques in the Methods section.*
☒	☐	A description of all covariates tested
☒	☐	A description of any assumptions or corrections, such as tests of normality and adjustment for multiple comparisons
☐	☒	A full description of the statistical parameters including central tendency (e.g. means) or other basic estimates (e.g. regression coefficient) AND variation (e.g. standard deviation) or associated estimates of uncertainty (e.g. confidence intervals)
☒	☐	For null hypothesis testing, the test statistic (e.g. *F*, *t*, *r*) with confidence intervals, effect sizes, degrees of freedom and *P* value noted<br>*Give P values as exact values whenever suitable.*
☒	☐	For Bayesian analysis, information on the choice of priors and Markov chain Monte Carlo settings
☒	☐	For hierarchical and complex designs, identification of the appropriate level for tests and full reporting of outcomes
☒	☐	Estimates of effect sizes (e.g. Cohen's *d*, Pearson's *r*), indicating how they were calculated

Our web collection on statistics for biologists contains articles on many of the points above.

Software and code

Policy information about availability of computer code

Data collection	Gel images were acquired using Typhoon 9400 Scanner Control Software v5.
Data analysis	Graphs were generated using Prism 5 (GraphPad). Data points were fitted to the Michaelis-Menten equation in Prism software.

For manuscripts utilizing custom algorithms or software that are central to the research but not yet described in published literature, software must be made available to editors/reviewers. We strongly encourage code deposition in a community repository (e.g. GitHub). See the Nature Research guidelines for submitting code & software for further information.

Data

Policy information about availability of data

All manuscripts must include a data availability statement. This statement should provide the following information, where applicable:

- Accession codes, unique identifiers, or web links for publicly available datasets
- A list of figures that have associated raw data
- A description of any restrictions on data availability

The source data underlying Figs. 1e, 1h, 3d, 4c, 4e, Supplementary Figs. 2, 4d, 5, and 8 are provided as a Source Data file. All other relevant data are available from the authors.

Field-specific reporting

Please select the one below that is the best fit for your research. If you are not sure, read the appropriate sections before making your selection.

☒ Life sciences ☐ Behavioural & social sciences ☐ Ecological, evolutionary & environmental sciences

Life sciences study design

All studies must disclose on these points even when the disclosure is negative.

Sample size	Triplicates are generally used in binding and enzyme kinetics assays.
Data exclusions	Due to the low activity of separase, our peptide cleavage assays generate low fluorescence signals, resulting in occasional brief fluctuations in the fluorescence signal. These results are excluded from the analysis.
Replication	All results are representative of multiple independent experiments.
Randomization	Not relevant to our studies.
Blinding	Not relevant to our studies.

Reporting for specific materials, systems and methods

We require information from authors about some types of materials, experimental systems and methods used in many studies. Here, indicate whether each material, system or method listed is relevant to your study. If you are not sure if a list item applies to your research, read the appropriate section before selecting a response.

Materials & experimental systems

n/a	Involved in the study	
☐	☒	Antibodies
☐	☒	Eukaryotic cell lines
☒	☐	Palaeontology
☒	☐	Animals and other organisms
☒	☐	Human research participants
☒	☐	Clinical data

Methods

n/a	Involved in the study	
☒	☐	ChIP-seq
☒	☐	Flow cytometry
☒	☐	MRI-based neuroimaging

Antibodies

Antibodies used	Anti-StrepII: StrepMAB-Classic-HRP, IBA GmbH<br>Anti-FLAG: Anti-FLAG M2 Monoclonal, Sigma.
Validation	Both antibodies are commercially produced and validated monoclonal antibodies directed against a specific peptide epitope and widely used for detection of highly expressed recombinant proteins.

Eukaryotic cell lines

Policy information about cell lines

Cell line source(s)	293T and U2OS cells were obtained from the neighboring laboratory of Ron Vale, which uses them for the same purpose.
Authentication	Not authenticated.
Mycoplasma contamination	Not tested.
Commonly misidentified lines (See ICLAC register)	None.
